# Supplementary material for: Oral Health-Related Quality of Life in Rare Disorders of Congenital Facial Weakness
Source: Int J Environ Res Public Health. 2024 May 13;21(5):615. doi: 10.3390/ijerph21050615 (PMC11121611; doi:10.3390/ijerph21050615)
Supplement: Supplementary file 1 [file ijerph-21-00615-s001.zip › ijerph-2961576-supplementary.docx]

**Supplementary Table S1.** Characteristics of the subjects who had undergone facial animation surgery.

| **Variable** | | **Facial Weakness Cohort: N (%)** |
| --- | --- | --- |
| **Total Subjects (N)** | | 10 |
| **Gender** | Male | 4 (40%) |
|  | Female | 6 (60%) |
| **Age** | Mean* (Years) | 39.84 |
|  | SD | 17.3 |
|  | Range | 11.4-64.2 |
| **Diagnosis** | MBS | 6 (60%) |
|  | HCFP | 1 (10%) |
|  | CFZ | 1 (10%) |
|  | TUBB3 | 0 |
|  | Other | 2 (20%) |

*At the time of completion of the OHIP-14 questionnaire.

**Supplementary Table S2.** Members of the Moebius Syndrome Research Consortium.

| **Name** | **Degree(s)** | **Preferred Email** | **Primary affiliation at time of research/referral** | **Additional affiliation(s)at time of research/referral** | **Current affiliation/s** |
| --- | --- | --- | --- | --- | --- |
|  |  |  |  |  |  |
| **Mt Sinai** |  |  |  |  |  |
| Ethylin W. Jabs | MD | [jabs.ethylin@mayo.edu](mailto:jabs.ethylin@mayo.edu) | Department of Genetics and Genomic Sciences, Icahn School of Medicine at Mount Sinai, New York, NY 10029, USA | Institute of Genetic Medicine, Johns Hopkins School of Medicine, Baltimore, MD 21205, USA | Department of Clinical Genomics, Mayo Clinic, Rochester, MN 55902, USA |
| Bryn D. Webb | MD | [bdwebb@wisc.edu](mailto:bdwebb@wisc.edu) | Department of Genetics and Genomic Sciences, Icahn School of Medicine at Mount Sinai, New York, NY 10029, USA | Department of Pediatrics, University of Wisconsin School of Medicine and Public Health, Madison, WI 53705, USA | Department of Pediatrics, University of Wisconsin School of Medicine and Public Health, Madison, WI 53705, USA |
| Zhongyang Zhang | PhD | zhongyang.zhang@mssm.edu | Department of Genetics and Genomic Sciences, Icahn School of Medicine at Mount Sinai, New York, NY 10029, USA |  | Department of Genetics and Genomic Sciences, Icahn School of Medicine at Mount Sinai, New York, New York 10029, USA |
| Ke Hao | ScD | [ke.hao@mssm.edu](mailto:ke.hao@mssm.edu) | Department of Genetics and Genomic Sciences, Icahn School of Medicine, New York, NY 10029, USA Mount Sinai, New York, NY 10029, USA |  | Department of Genetics and Genomic Sciences, Icahn School of Medicine at Mount Sinai, New York, NY 10029, USA |
| Janet C. Rucker | MD | [janet.rucker@nyumc.org](mailto:janet.rucker@nyumc.org) | Bernard A. and Charlotte Marden Department of Neurology, New York University School of Medicine, New York, NY 10017, USA | Department of Neurology, Icahn School of Medicine at Mount Sinai, New York, NY 10029, USA | Bernard A. and Charlotte Marden Department of Neurology, New York University School of Medicine, New York, NY 10017; Department of Neurology, Icahn School of Medicine at Mount Sinai, New York, NY 10029, USA |
| Tamiesha Frempong | MD | [tamiesha.frempong@mssm.edu](mailto:tamiesha.frempong@mssm.edu) | New York Eye and Ear Infirmary of Mount Sinai, New York, NY 10029, USA |  | New York Eye and Ear Infirmary of Mount Sinai, New York, NY 10029, USA |
| Monica Erazo | MS | [monica.erazo@mssm.edu; monerazo@gmail.com](mailto:monica.erazo@mssm.edu) | Icahn School of Medicine at Mount Sinai \| MSSM · Department of Genetics and Genomic Sciences, New York, NY 10029, USA |  | Icahn School of Medicine at Mount Sinai \| MSSM · Department of Genetics and Genomic Sciences, New York, NY 10029, USA |
|  |  |  |  |  |  |
|  |  |  |  |  |  |
| **Boston-Children's Hospital - Harvard** |  |  |  |  |  |
| Elizabeth C Engle | MD | elizabeth.engle@childrens.harvard.edu | Department of Neurology, Pathology, Ophthalmology, F.M. Kirby Neurobiology Center, Boston Children’s Hospital, Boston, MA 02115, USA | Howard Hughes Medical Institute, Chevy Chase, MD 20815, USA | Department of Neurology, Pathology, Ophthalmology, F.M. Kirby Neurobiology Center, Boston Children’s Hospital, Boston, Massachusetts 02115, USA; Howard Hughes Medical Institute, Chevy Chase, MD 20815, USA |
| Alan P. Tenney | PhD | Alan.Tenney@childrens.harvard.edu | Department of Neurology, Boston Children’s Hospital, Boston, MA 02115, USA | F.M. Kirby Neurobiology Center, Boston Children’s Hospital, Boston, MA 02115, USA | Department of Neurology, Boston Children’s Hospital, Boston, MA 02115, USA; F.M. Kirby Neurobiology Center, Boston Children’s Hospital, Boston, MA 02115, USA |
| Brenda Barry | MS | brenda.barry2@childrens.harvard.edu | Department of Neurology, Boston Children’s Hospital, Boston, MA 02115, USA | Howard Hughes Medical Institute, Chevy Chase, MD 20815, USA | Department of Neurology, Boston Children’s Hospital, Boston, MA 02115, USA |
| Wai-Man Chan | MS | Wai-Man.Chan@childrens.harvard.edu | Department of Neurology, Boston Children’s Hospital, Boston, MA 02115, USA | Howard Hughes Medical Institute, Chevy Chase, MD 20815, USA | Department of Neurology, Boston Children’s Hospital, Boston, MA 02115, USA |
| Caroline D. Robson | MD | Caroline.Robson@childrens.harvard.edu | Department of Radiology, Boston Children’s Hospital, Boston, MA 02115, USA | Harvard Medical School, Boston, MA 02115, USA | Department of Neurology, Boston Children’s Hospital, Boston, MA 02115, USA |
| Silvio Alessandro Di Gioia | PhD | alessandro.digioia2@gmail.com | Department of Neurology, Boston Children’s Hospital, Boston, MA 02115, USA | F.M. Kirby Neurobiology Center, Boston Children’s Hospital, Boston, MA 02115, USA |  |
| David G. Hunter | MD | [david.hunter@childrens.harvard.edu](mailto:david.hunter@childrens.harvard.edu) | Department of Ophthalmology, Boston Children’s Hospital, Boston, MA 02115, USA | Department of Ophthalmology, Harvard Medical School, Boston, MA 02115, USA | Department of Ophthalmology, Boston Children’s Hospital, Boston, MA 02115, USA; Department of Ophthalmology, Harvard Medical School, Boston, MA 02115, USA |
| Sarah E. Mackinnon |  | [Sarah.Mackinnon@CHILDRENS.HARVARD.EDU](mailto:Sarah.Mackinnon@CHILDRENS.HARVARD.EDU) | Department of Ophthalmology, Boston Children’s Hospital, Boston, MA 02115, USA |  | Department of Ophthalmology, Boston Children’s Hospital, Boston, MA 02115, USA |
| Matthew Rose | MD | [Matthew.Rose@childrens.harvard.edu](mailto:Matthew.Rose@childrens.harvard.edu) | Departments of Neurology, Pathology, F.M. Kirby Neurobiology Center, Boston Children’s Hospital, Boston, MA 02115, USA | Department of Pathology, Brigham and Women’s Hospital, Boston, MA 02115, USA | Departments of Neurology, Pathology, F.M. Kirby Neurobiology Center, Boston Children’s Hospital, Boston, MA 02115, USA; Department of Pathology, Brigham and Women’s Hospital, Boston, MA 02115, USA |
|  |  |  |  |  |  |
|  |  |  |  |  |  |
| **NIH** | |  |  |  |  |
| Irini Manoli | MD, PhD | [manolii@mail.nih.gov](mailto:manolii@mail.nih.gov) | Medical Genomics and Metabolic Genetics Branch, National Human Genome Research Institute, Bethesda, MD 20892, USA | Metabolic Medicine Branch, National Human Genome Research Institute, National Institutes of Health, Bethesda, MD 20892, USA | Medical Genomics and Metabolic Genetics Branch, National Human Genome Research Institute, Bethesda, MD 20892, USA |
| Carol Van Ryzin | CPNP | [carol.vanryzin@nih.gov](mailto:carol.vanryzin@nih.gov) | Medical Genomics and Metabolic Genetics Branch, National Human Genome Research Institute, Bethesda, MD 20892, USA | Metabolic Medicine Branch, National Human Genome Research Institute, National Institutes of Health, Bethesda, MD 20892, USA | Medical Genomics and Metabolic Genetics Branch, National Human Genome Research Institute, Bethesda, MD 20892, USA |
| Flavia Facio | MS | flavia.facio@nih.gov | Medical Genomics and Metabolic Genetics Branch, National Human Genome Research Institute, Bethesda, MD 20892, USA |  |  |
| Tanya J. Lehky | MD | lehkyt@ninds.nih.gov | Electromyography Section under the Office of the Clinical Director, National Institute of Neurological Disorders and Stroke, Bethesda, MD 20892, USA |  | NA |
| Camilo Toro | MD | [toroc@mail.nih.gov](mailto:toroc@mail.nih.gov) | NIH Undiagnosed Diseases Program, Common Fund, National Human Genome Research Institute, National Institutes of Health, Bethesda, MD 20892, USA |  | NIH Undiagnosed Diseases Program, Common Fund, National Human Genome Research Institute, National Institutes of Health, Bethesda, MD 20892, USA |
| Andrea L. Gropman | MD | [AGropman@childrensnational.org](mailto:AGropman@childrensnational.org) | George Washington University and Children’s National Medical Center, Washington, DC 20037, USA | Children's National Medical Center, Division of Developmental Pediatrics, Washington DC 20010, USA | Children's National Medical Center, Division of Developmental Pediatrics, Washington DC 20010, USA |
| Christopher Zalewski | PhD, Au.D. | zalewski@nidcd.nih.gov | Audiology Unit, Otolaryngology Branch, National Institute on Deafness and Other Communication, Disorders, Bethesda, MD 20892, USA |  | Audiology Unit, Otolaryngology Branch, National Institute on Deafness and Other Communication, Disorders, Bethesda, MD 20892, USA |
| Kelly A King | PhD, Au.D. | kingke@nidcd.nih.gov | Audiology Unit, Otolaryngology Branch, National Institute on Deafness and Other Communication Disorders, Bethesda, MD 20892, USA |  | Audiology Unit, Otolaryngology Branch, National Institute on Deafness and Other Communication Disorders, Bethesda, MD 20892, USA |
| Carmen C. Brewer | PhD | [brewerc@nidcd.nih.gov](mailto:brewerc@nidcd.nih.gov) | Chief Research Audiologist, Audiology Unit, Otolaryngology Branch, National Institute of Deafness and other Communications Disorders, Bethesda, MD 20892, USA |  | Chief Research Audiologist, Audiology Unit, Otolaryngology Branch, National Institute of Deafness and other Communications Disorders, Bethesda, MD 20892, USA |
| Audrey Thurm | PhD | athurm@mail.nih.gov | Pediatrics and Developmental Neuroscience Branch, National Institute of Mental Health, Bethesda, MD 20892, USA |  | Pediatrics and Developmental Neuroscience Branch, National Institute of Mental Health, Bethesda, MD 20892, USA |
| Joseph Snow | PhD | josephsnow@mail.nih.gov | Office of the Clinical Director, National Institute of Mental Health, Bethesda, MD 20892, USA |  | Office of the Clinical Director, National Institute of Mental Health, Bethesda, MD 20892, USA |
| Scott M. Paul | MD | [SPaul@cc.nih.gov](mailto:SPaul@cc.nih.gov) | Rehabilitation Medicine Department, NIH Clinical Center, Bethesda, MD 20892, USA |  | Rehabilitation Medicine Department, NIH Clinical Center, Bethesda, MD 20892, USA |
|  |  |  |  |  |  |
| Francis S. Collins | MD, PhD | [francis.collins@nih.gov](mailto:francis.collins@nih.gov) | Medical Genomics and Metabolic Genetics Branch, National Human Genome Research Institute, Bethesda, MD 20892, USA |  | Medical Genomics and Metabolic Genetics Branch, National Human Genome Research Institute, Bethesda, MD 20892 USA; Center for Precision Health Research, National Human Genome Research Institute, Bethesda, MD 20892, USA |
| Narisu Narisu | PhD | narisu@mail.nih.gov | Medical Genomics and Metabolic Genetics Branch, National Human Genome Research Institute, National Institutes of Health, Bethesda, MD 20892, USA |  | Medical Genomics and Metabolic Genetics Branch, National Human Genome Research Institute, National Institutes of Health, Bethesda, MD 20892, USA |
| Lori L. Bonnycastle | PhD | lbonnyca@nih.gov | Medical Genomics and Metabolic Genetics Branch, National Human Genome Research Institute, Bethesda, MD 20892, USA |  | Medical Genomics and Metabolic Genetics Branch, National Human Genome Research Institute, Bethesda, MD 20892, USA |
| Amy J. Swift | MS | amy.swift@nih.gov | Medical Genomics and Metabolic Genetics Branch, National Human Genome Research Institute, Bethesda, MD 20892, USA |  | Medical Genomics and Metabolic Genetics Branch, National Human Genome Research Institute, Bethesda, MD 20892, USA |
| Peter S. Chines | PhD | Deceased | Medical Genomics and Metabolic Genetics Branch, National Human Genome Research Institute, Bethesda, MD 20892, USA |  | Medical Genomics and Metabolic Genetics Branch, National Human Genome Research Institute, Bethesda, MD 20892, USA |
|  |  |  |  |  |  |
| Carlo Pierpaoli | MD, PhD | cp1a@nih.gov | Section on Tissue Biophysics and Biomimetics, National Institute of Child Health and Human Development, Bethesda, MD 20892, USA | Quantitative Medical Imaging Section, National Institute of Biomedical Imaging and Bioengineering | Section on Tissue Biophysics and Biomimetics, National Institute of Child Health and Human Development, Bethesda, MD 20892, USA |
| Neda Sadeghi | PhD | neda.sadeghi@nih.gov, nedasadeghi@icloud.com | Section on Tissue Biophysics and Biomimetics, National Institute of Child Health and Human Development, Bethesda, MD 20892, USA | Quantitative Medical Imaging Section, National Institute of Biomedical Imaging and Bioengineering | Section on Tissue Biophysics and Biomimetics, National Institute of Child Health and Human Development, Bethesda, MD 20892, USA |
| Brian Brooks | MD, PhD | [brooksb@nei.nih.gov](mailto:brooksb@nei.nih.gov) | Investigator, Ophthalmic Genetics & Visual Function Branch, National Eye Institute, Bethesda, MD 20892, USA |  | Investigator, Ophthalmic Genetics & Visual Function Branch, National Eye Institute, Bethesda, MD 20892, USA |
| Edmond J. FitzGibbon | MD | [ejf@lsr.nei.nih.gov](mailto:ejf@lsr.nei.nih.gov) | National Eye Institute, Bethesda, MD 20892, USA |  | National Eye Institute, Bethesda, MD 20892, USA |
| Janice S. Lee | DDS, MD, FACS | [janice.lee@nih.gov](mailto:janice.lee@nih.gov) | National Institute of Dental and Craniofacial Research, Bethesda, MD 20892, USA |  | National Institute of Dental and Craniofacial Research, Bethesda, MD 20892, USA |
| Japee Shruti | PhD | [japees@mail.nih.gov](mailto:japees@mail.nih.gov) | Lab of Brain and Cognition, National Institute of Mental Health, Bethesda, MD 20892, USA |  | Lab of Brain and Cognition, National Institute of Mental Health, Bethesda, MD 20892, USA |
